# Supplementary material for: Multiple Mycotoxins in Kenyan Rice
Source: Toxins (Basel). 2021 Mar 11;13(3):203. doi: 10.3390/toxins13030203 (PMC7998506; doi:10.3390/toxins13030203)
Supplement: Supplementary file 1 [file toxins-13-00203-s001.pdf]

Supplementary Materials: Multiple Mycotoxins in Kenyan Rice

Samuel K. Mutiga, J. Musembi Mutuku, Vincent Koskei, James Kamau Gitau, Fredrick Ng'ang'a, Joyce Musyoka, George N. Chemining'wa and Rosemary Murori

Table S1. LC-MS/MS optimized multiple reaction monitoring (MRM) parameters for analytes including retention time (RT), precursor ions, product ions and respective collision energies. Identification and quantification conducted in Shimadzu 8050 LC-MS/MS system equipped with an ion electrospray ionization source operating in both positive and negative ionization modes. The equipment was set as follows: Nebulizing gas, 2.5 L/min; Drying gas, 10 L/min; heating gas, 10 L/min; Interface voltage, 4 kV; Temperatures (Interface, 300 °C; Heating block, 400 °C; Desolvation line 200 °C) and CID Gas flow 270 KPA).

Mycotoxin	Retention Time	Molecular Weight	Ionisation Mode	Precursor Ion	Product Ion	Q1 Pre-bias	Collision Energy	Q3 Pre-bias
Aflatoxin G2	6.275	330.292	ESI+	330.80 $[M+H]^+$	313.15/245.10	−25.0/−25.0	−25.0/−31.0	−23.0/−27.0
Aflatoxin G1	6.433	328.276	ESI+	328.80 $[M+H]^+$	243.15/311.15	−30.0/−30.0	−27.0/−23.0	−26.0/−22.0
Aflatoxin B1	6.804	312.2798	ESI+	312.80 $[M+H]^+$	285.15/241.15	−30.0/−30.0	−22.0/−38.0	−20.0/−26.0
Aflatoxin B2	6.684	314.29	ESI+	314.80 $[M+H]^+$	287.20/259.15	−24.0/−24.0	−26.0/−29.0	−21.0/−28.0
Fumonisin B1	7.302	721.83	ESI+	722.20 $[M+H]^+$	352.40/334.40	−38.0/−38.0	−49.0/−28.0	−19.0/−20.0
Fumonisin B2	6.684	705.83	ESI+	706.70 $[M+H]^+$	336.45/354.45	−26.0/−26.0	−39.0/−35.0	−25.0/−25.0
Fumonisin B3	7.577	705.839	ESI+	706.85 $[M+H]^+$	109.00/318.30/133.10	−20.0/−22.0/−28.0	−48.0/−14.0/−42.0	−30.0/−24.0/−12.0
HT2	7.231	424.49	ESI+	446.90 $[M+H]^+$	345.25/285.25	−23.0/−23.0	−19.0/−22.0	−25.0/−21.0
T2	7.562	466.527	ESI+	484.00 $[M+H]^+$	215.20/185.15	−25.0/−25.0	−21.0/−23.0	−24.0/−21.0
Diacetoxyscirpenol	6.678	366.41	ESI+	384.40 $[M+H]^+$	307.25/247.10	−19.0/−19.0	−13.0/−15.0	−22.0/−18.0
Ochratoxin A	7.906	403.81	ESI+	404.00 $[M+H]^+$	239.00/358.05	−29.0/−29.0	−24.0/−14.0	−26.0/−26.0
Citrinin	7.015	250.25	ESI+	251.25 $[M+H]^+$	233.00/205.10/91.20	−13.0/−13.0/−13.0	−17.0/−26.0/−48.0	−26.0/−22.0/−19.0
Sterigmatocystin	8.239	324.284	ESI+	325.30 $[M+H]^+$	310.05/281.05/253.05	13.0/−13.0/−13.0	−25.0/−37.0/−44.0	−22.0/−30.0/−28.0

Table S2. LC-MS/MS method validation and performance characteristics: linearity, spike recovery, matrix effects, and analytical limits.

	Spike Recovery Tests			Signal Suppression/ Enhancement (SSE)	Final Methods Prediction Model		
**Mycotoxin**	**Spiked Level (ng/g)**	**Average Recovery (%)**	**RSD (%)**	**SSE (%)**	**R^2**	**LOD**	**LOQ**
Aflatoxin G1	4.21, 10.5, 21.05	95	13	141	0.99	0.05	0.17
Aflatoxin G2	4.21, 10.5, 21.05	102	5	95	0.99	0.10	0.34
Aflatoxin B1	4.18, 20.45, 20.9	103	12	144	0.99	0.04	0.13
Aflatoxin B2	4.196, 10.49, 20.98	52	9	108	0.98	0.07	0.25
Fumonisin B1	10.02, 25.05, 50.1	82	12	127	0.96	1.53	5.10
Fumonisin B2	10.02, 25.05, 50.1	105	14	137	0.99	0.15	0.49
Fumonisin B3	10.2, 25.53, 51.06	100	9	133	0.97	0.67	2.25
HT2	2.04, 5.1, 10.2	78	8	128	0.85	0.03	0.11
T2	2.04,5.1, 10.2	102	5	124	0.80	0.01	0.05
Diacetoxyscirpenol	2.04, 5.1, 10.2	89	12	88	0.98	0.05	0.18
Ochratoxin A	10.08, 25.2, 50.4	99	8	99	0.97	0.19	0.62
Citrinin	20.58, 51.45, 102.9	64	15	132	0.98	0.10	0.33
Sterigmatocystin	10.02, 25.05, 50.1	80	3	107	0.98	0.03	0.09

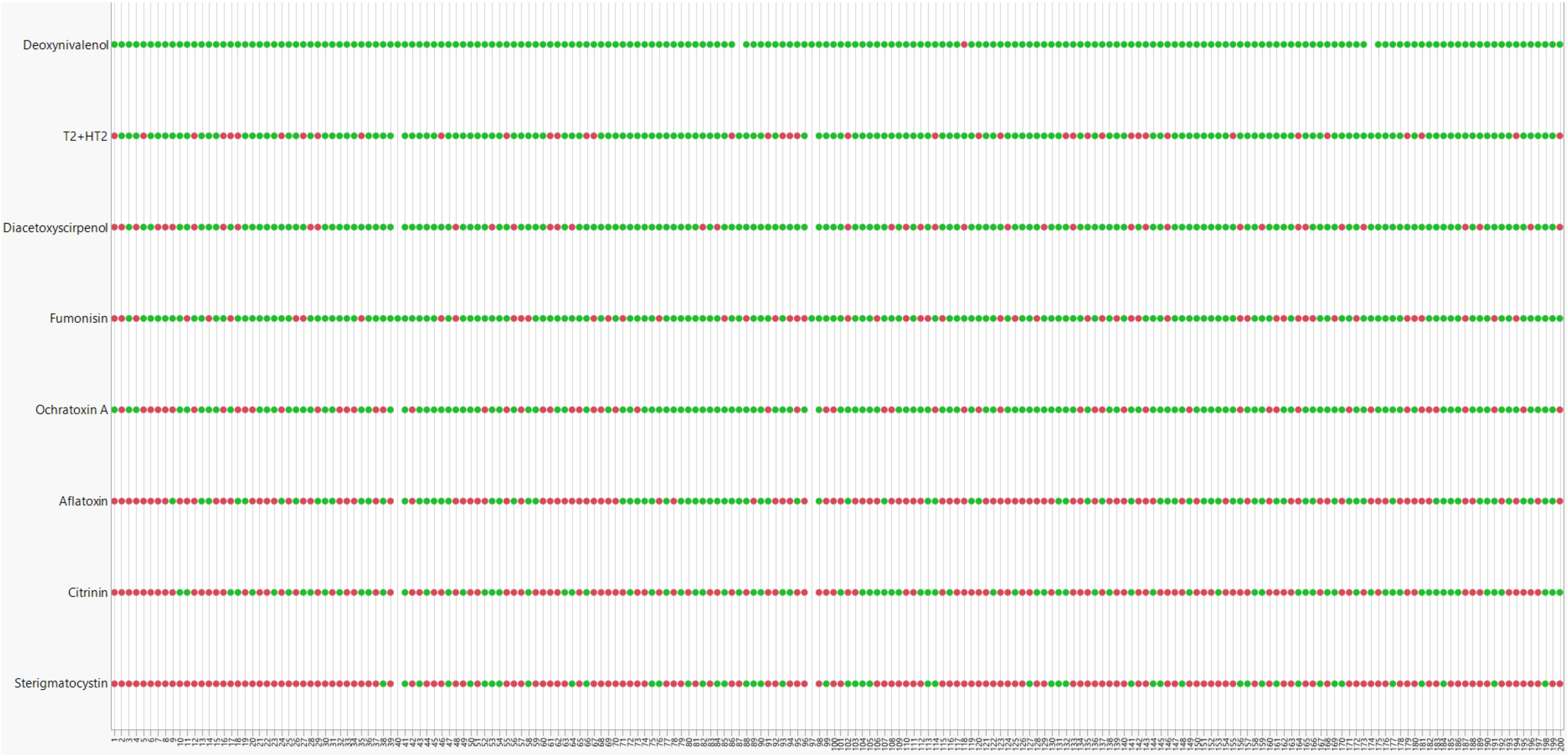

Figure S1. Occurrence of multiple mycotoxins in rice from Kirinyaga County, Kenya. Colors: Green = mycotoxin is present; red = mycotoxin is absent. Limit of detection is based on minimum detectable concentrations of individual toxins as optimized using the LC-MS/MS or ELISA (for deoxynivalenol; Helica Biosystems, Inc., Santa Ana, CA, USA).
